# Prevalence of Lower Cross Syndrome in Housemaids

**DOI:** 10.7759/cureus.57425

**Published:** 2024-04-01

**Authors:** Ghanishtha Burile, Pratik Phansopkar, Nikita S Deshmukh

**Affiliations:** 1 Musculoskeletal Physiotherapy, Ravi Nair Physiotherapy College, Datta Meghe Institute of Higher Education and Research, Wardha, IND

**Keywords:** lower limb alignment, postural changes, weak front muscle, tight, housemaids, muscular imbalance, low back pain, prevalence

## Abstract

Introduction

Lower cross syndrome, also known as pelvic crossed syndrome, occurs if there is inadequate muscle strength, leading to an imbalance in the lower extremities. This condition is characterized by the weakening and tightening of muscle groups on the anterior and posterior aspects of the body. Mostly, there is weakness in the abdominal muscles, gluteus maximus, and gluteus medius, while there is tightness in the hip flexor muscle groups. There are various studies investigating musculoskeletal disorders across different professions, but there is no research on the prevalence of lower cross syndrome among housemaids. Housemaids frequently report complaints of joint pain and exhibit specific postural changes such as anterior pelvic tilt, increased lumbar lordosis, and lower back pain. Therefore, this research aims to fill this gap by determining the prevalence of lower cross syndrome within the housemaid profession. The study aims to find out the prevalence of lower cross syndrome among housemaids.

Methodology

A total of 75 housemaids between the ages of 35 and 50 years complaining of pain in the lower back were included in the study, and the housemaids with recent surgical histories and cognitive impairments were excluded. The evaluation was done by measuring the strength and range of motion (ROM) of the affected muscles. Outcome measures include the length of the iliopsoas muscle, measurement of the spinal extensor muscle, and strength of the gluteus maximus muscle to identify which structures are tight or weak.

Result

Statistical changes were observed in the housemaids' population to check tight and weak structures using all outcome measures. According to the visual analogue scale (VAS), the pain was found to have a standard deviation of 5.39 ± 1.26 (3-8). The length of the iliopsoas muscle on the right and left sides had a t-value of 1.51 (p = 0.13), and the length of lumbar extensors had a standard deviation of 5.39 ± 1.26 (3-8).

Conclusion

In our study, the conclusion was found that housemaids who are working continuously for long periods without maintaining good ergonomics are prone to lower cross syndrome. It indicates a strong need for further research on the management of symptoms in such a population (housemaids) to prevent chronic musculoskeletal illness.

## Introduction

One of the most prevalent conditions linked to postural imbalance is low back pain. Among 84% of individuals who reported having low back pain, 23% eventually developed chronic low back pain (CLBP) [1]. Postural imbalance can vary depending on spinopelvic parameters and may also be observed based on the lower muscular segment, such as in the lower cross syndromes [2]. Pelvic cross syndrome occurs due to muscular imbalances within the pelvic joint and muscle group. This imbalance manifests as weakened abdominal muscles and tightened hip flexors, leading to alterations in postural alignment [3]. In the research conducted on the angle of the lumbopelvic-hip junction in housemaids with complaints of low back pain and lower cross syndrome in housemaids, it was found that sitting upright and freestyle for long hours can cause muscular imbalance that can further lead to backache [4]. Due to reduced abdominal (core) muscle strength, CLBP is very common. In CLBP, there is a loss of the ability of the core muscles to inhibit excessive movement at the lumbar spine, which is known as control impairment [5]. Repetitive bending activities, reaching behind, twisting activities, wrist-bending, kneeling, stooping, and squatting, all these activities increase stress on supporting structures and stand out as the primary causes of poor posture, therefore negatively impacting physical activity [6,7].

Research in some articles indicates that the prepatellar bursa, positioned between the patella and subcutaneous tissue, emerges as the most frequently affected bursa of the knee. It is the second most commonly impacted bursa overall, trailing only the olecranon bursa. This bursa is often subjected to repetitive kneeling and is informally called a housemaid's or carpenter's knee [8]. Alteration in the pelvis affects lumbopelvic complex functions, which govern body movements. The combination of weak lower abdominal muscles and tight hip flexors causes the pelvis to rotate more posteriorly and lessen lumbar lordosis [9]. The "powerhouse," commonly described as the core muscle, serves as the foundation or "engine" for all limb movements. Strengthening of the core muscles through exercises aimed at stabilizing the spine aids in maintaining and enhancing posture. Functional core training allows for the practice of movements by optimizing stability and mobility for activities of daily living (ADLs) [10].

In some studies, it was found that women were more likely than men to develop lower cross syndrome [11]. Certain studies show that specific muscle groups, such as the hip flexors and adductors, or the shoulder protractors, demonstrate increased tonic activation during infant development, rendering them more susceptible to shortening and facilitation. Lower cross syndrome is considered the "mother" syndrome, with upper-cross syndrome emerging as a result of its occurrence [12,13]. If the individual has strong core muscle strength, then good alignment of the spine can be maintained for a prolonged period of time [14]. Females wearing high-heeled shoes can lead to a prevalent muscular imbalance in the lower torso. This imbalance mostly involves the hip flexors, including the iliopsoas, and the upper anterior thigh muscles, such as the rectus femoris and sartorius. These anterior hypertonic muscle groups are directly associated with hyperlordosis [15]. There was no correlation found between lumbar lordosis and pelvic inclination in a standing position and abdominal muscular force, according to a study done on people with lumbar lordosis and pelvic inclination who had persistent low back pain [16]. The typical lumbar curve can be altered, leading to a decrease in stooping or sitting or an increase in standing straight, primarily because of the opposing side's trunk-thigh muscles' limited length [17,18].

If there is increased abdominal pressure, it contributes to lumbar spinal stability, as anticipated by a buckling model analysis. However, contracting the transversus abdominis or oblique muscles did not significantly impact the level of spinal stability [19,20]. Imagery exercises significantly altered the electromyographic (EMG) responses of the gluteus maximus and rectus femoris muscles, which are two lumbopelvic muscles. On the other hand, its impact on the degree and strength of lumbar lordosis was insignificant. When it came to altering the EMG activity of the lumbopelvic muscles and the strength of the abdominal and gluteus maximus muscles, the combined exercise was just as successful as the active exercise [21]. Muscle energy techniques (MET) are one of the treatment options for managing lower cross syndrome-related persistent low back pain in terms of pain and disability, reduced lumbar ROM, and some degrees of lumbar lordosis [22]. Compared to the supine position, the internal oblique muscle was substantially more active when sitting. Standing resulted in significantly more activity in the external and internal oblique abdominals than sitting did. The ideal crossing of the legs when seated reduces the activity of the oblique abdominals significantly (either upper legs crossed or ankle on knee) [23,24].

Every profession has certain musculoskeletal demands. Moreover, if these musculoskeletal demands are not fulfilled or compromised because of any musculoskeletal disorder, work efficiency and productivity in ones professional life are reduced and hampered along with the ADLs. Hence, the above study was designed to find the occurrence of lower cross syndrome in housemaids, who often ignore the symptoms until ADLs are affected.

## Materials and methods

After receiving ethical authorization and approval from the Institutional Ethical Committee of Datta Meghe Institute of Higher Education and Research(DU) with Ref. No. (DMIMS(DU)/IEC/2022/917), this research was conducted in local communities and at Acharya Vinobha Bhave Rural Hospital, Sawangi, Wardha, Maharashtra. In the study, 75 housemaids between the age group of 35 and 50 years were included who fit the inclusion and exclusion criteria. Informed consent was taken before taking the assessment. The inclusion criteria include working as a housemaid with complaints of pain in the lower back. Exclusion criteria include trauma, recent surgical history, or cognitive impairments.

Procedure

**Figure 1:**
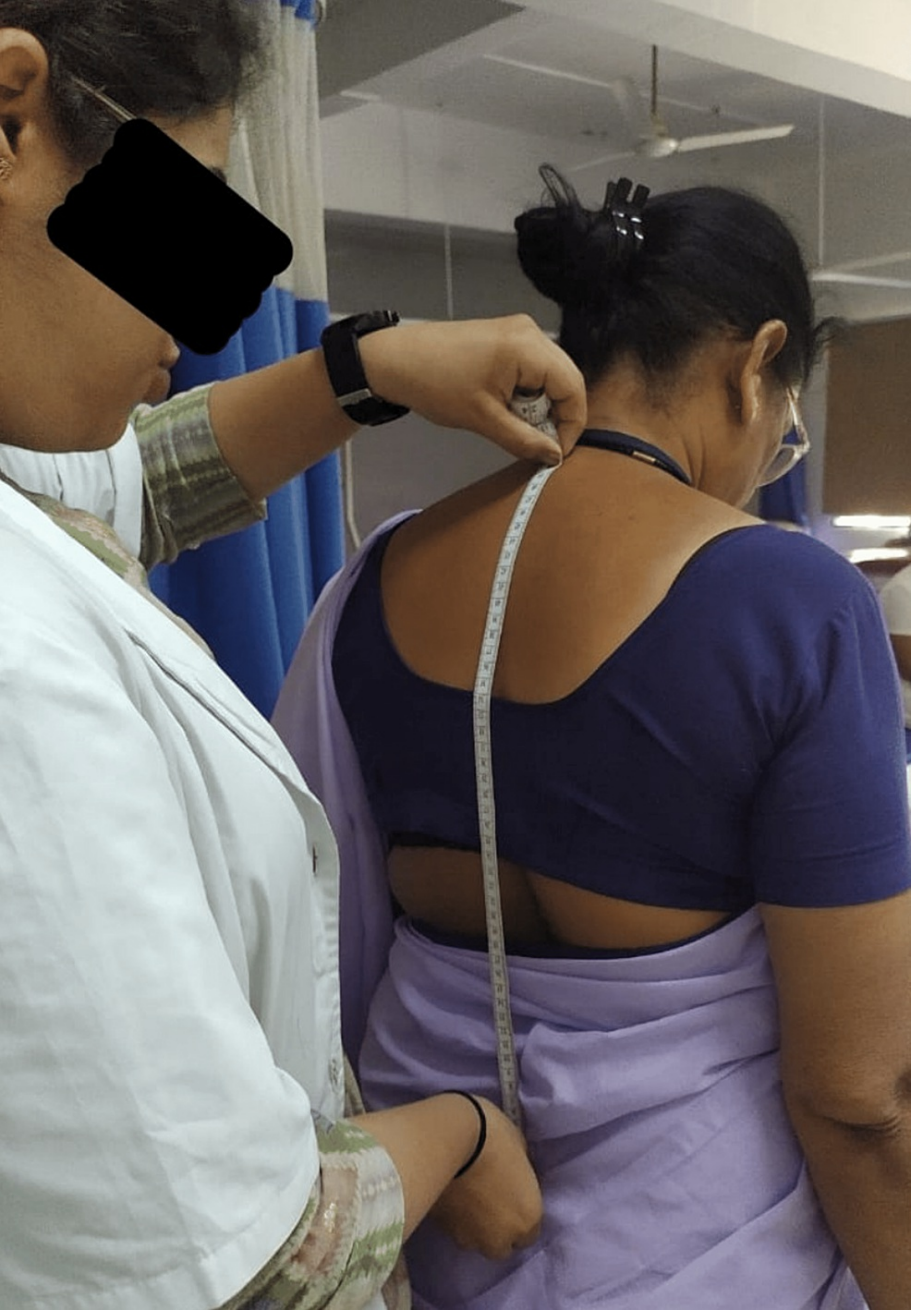
Measuring the length of the lumbar extensors using a measuring tape by the therapist

**Figure 2:**
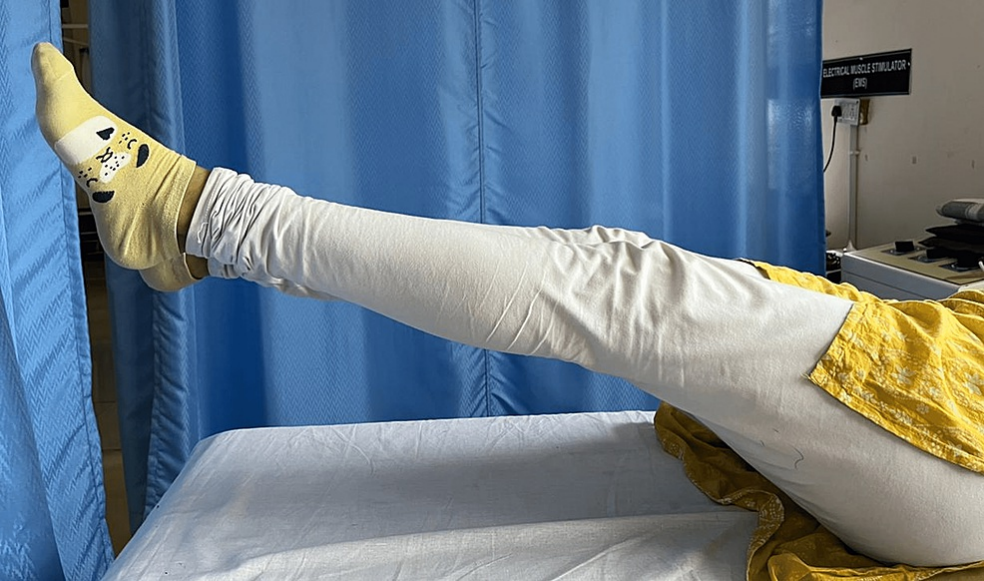
Assessment of the lower abdominal MMT MMT: Manual muscle testing

**Figure 3:**
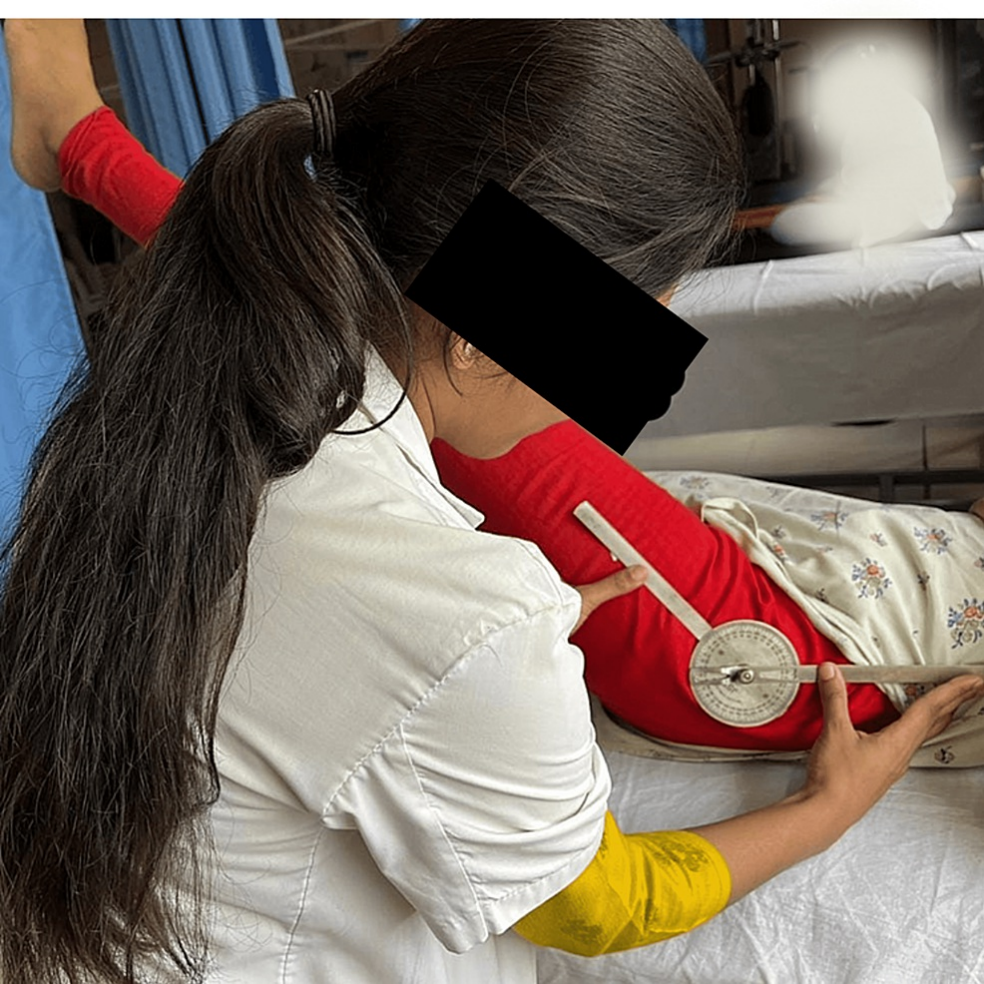
Therapist measuring the length of the iliopsoas muscle

All housemaids underwent screening according to the inclusion and exclusion criteria. Only those housemaids meeting the inclusion criteria were included in the study, while those with cognitive impairments and a history of trauma were excluded. A total of 75 housemaids participated independently in the study. They were provided with back stretches and warm-up exercises, involving 10 repetitions of side flexion and rotation. Subsequently, measurements were taken for the length of the muscles of the lumbar spine, range of motion (ROM) of the iliopsoas muscle, and strength of the abdominal muscles. To measure the length of the spinal extensor muscles, a measuring tape was employed. Initially, the spinous processes of the C7 and S1 vertebrae were marked using a marker, and then, the distance between these points was measured with the tape, representing the gap between the spinous processes. Throughout this process, participants were instructed to maintain a straight posture while flexing and rotating the cervical, thoracic, and lumbar spines to 0° as shown in Figure 1. The normal muscle length was defined as 10 cm. To assess the strength of the lower abdominal muscles, participants were instructed to lie in a supine position with their hips and knees extended, as mentioned in Figure 2. The strength of the gluteus maximus, in conjunction with the lower abdomen muscles, was evaluated using the Kendall grading system. Grade 6 (fair+) indicated the ability to elevate legs to approximately a 60° angle. Grade 8 (good) demonstrated the capacity to maintain a flat lower back while flexing the lower limbs to 30°. Grade 10 (normal) signified the capability to keep the lower back flat while lowering the legs to the level of the table. In Figure 3, the bilateral iliopsoas length was measured utilizing a goniometer. The goniometer was positioned with its axis at the greater trochanter, and the moving arm was aligned along the lateral midline of the femur, while the stationary arm was parallel to the lateral midline of the pelvis. This procedure was performed bilaterally. If the hip extension angle remained below 15°, it indicated iliopsoas tightness.

In Figure 1, the length of the lumbar extensors of the housemaid was measured.

In Figure 2, assessment of the lower abdominal manual muscle testing (MMT) was done to find out the strength of the abdominal muscles. 

In Figure 3, the therapist measures the length of the iliopsoas muscle using a goniometer. 

## Results

**Figure 4:**
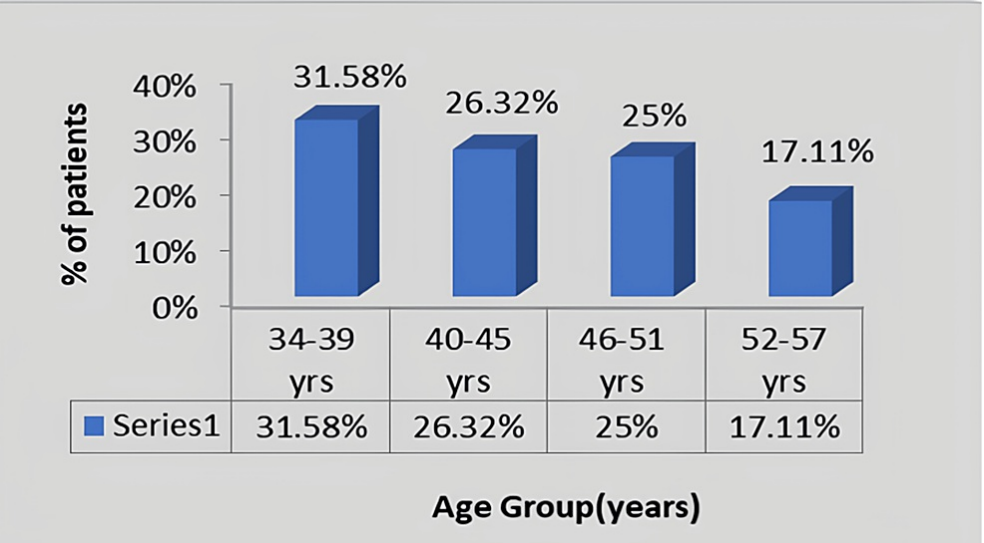
Graphical presentation of the distribution of the participants according to their age in years

In Figure 4, the participants were distributed according to their age group. A total of 75 housemaids were included; among which, 31.58% were between the ages of 34 and 39 years, 26.32% were between the ages of 40 and 45 years, 25% were between the ages of 46 and 51 years, and 17.11 % were between the ages of 52 and 57 years. 

**Figure 5:**
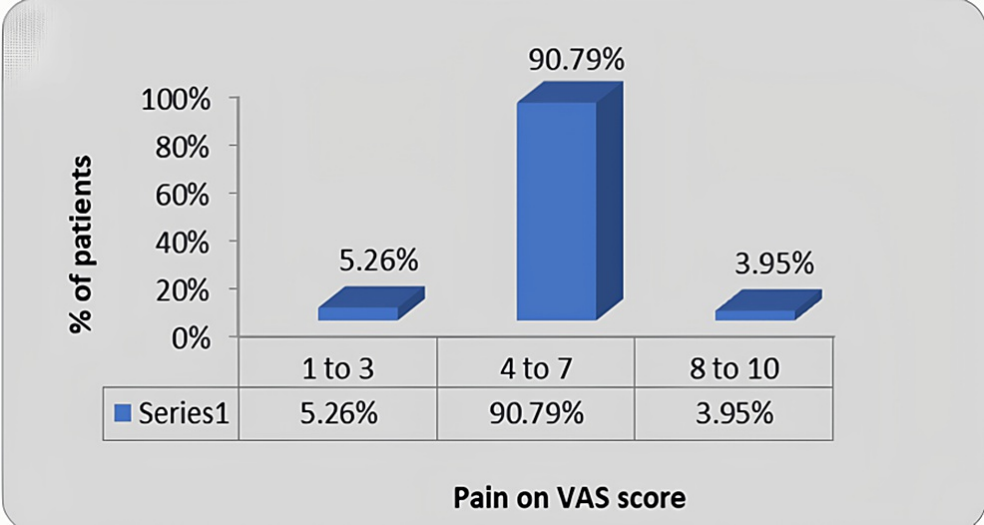
Graphical presentation for pain assessment using VAS VAS: Visual analogue scale

In Figure 5, pain assessment was done using a visual analogue scale (VAS). Among which, 5.26% of the participants rated pain between 1 and 3, 90.79% of the participants rated pain between 4 and 7, and 3.95% of the participants rated pain as between 8 and 10. 

**Figure 6:**
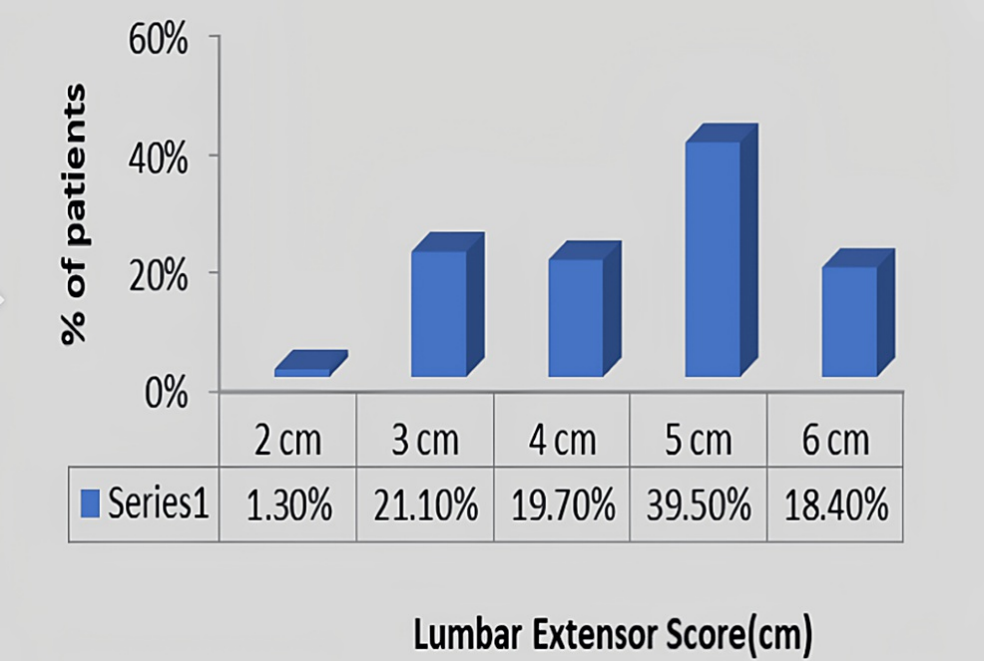
Graphical representation on the assessment of the length of the lumbar extensors in housemaids

In Figure 6, assessment of the length of the lumbar extensors in 75 housemaids was done. In 1.30% of the participants, the length of the lumbar extensors was 2 cm; in 21.10% of the participants, it was 3 cm; in 19.70% of the participants, it was 4 cm; in 39.50% of the participants, it was 5 cm; and in 18.40% of the participants, it was 6 cm.

In Table 1, the mean error for the ROM of the iliopsoas muscles of the right and left sides was 0.25 ± 1.44, indicating that the left side was more affected than the right side.

**Table 1:** Comparison of the iliopsoas ROM bilaterally ROM: Range of motion

	Mean	N	Std. deviation	Std. error mean	Mean difference	t-value
Right side	8.76	76	2.57	0.29	0.25 ± 1.44	1.51; P = 0.13, NS
Left side	9.01	76	2.65	0.30		

In Table 2, the lower abdominal strength was measured using the Kendall grading system. A total of 76 housemaids were included; among which, 64.47% of the housemaids had a fair grade, while 35.53% had good abdominal strength.

**Table 2:** Lower abdominal muscle strength using the Kendall grading system MMT: Manual muscle testing

MMT abdominal	No. of patients	Percentage
Fair	49	64.47
Good	27	35.53
Total	76	100

Statistical analysis was done by using descriptive and inferential statistics using students' paired t-tests, and the software used in the analysis was IBM SPSS Statistics for Windows, Version 27.0 (Released 2020; IBM Corp., Armonk, New York, United States), and p < 0.05 is considered as the significance level.

## Discussion

Housemaids who work for prolonged periods without looking at their posture while working can experience low back pain. Lower cross syndrome leads to muscular imbalance in muscle groups like hip flexor tightness and weakness at the abdominals and gluteus, which alters the kinematic chain. As a result, this study aims to find the prevalence of weak and tight structures that are involved in the lower cross syndrome in housemaids. Bhore et al. researched on the prevalence of core muscle weaknesses in bank employees [25]. According to the research, the average rate of core weakness among bank workers was found to be 72.73%; the age range of 45-50 years had the highest percentage of core weakness (23.6%), which was comparatively greater than that of other age ranges [26]. Strengthening of the core muscles effectively decreases pain and promotes dynamic balance in female subjects with patellofemoral pain syndrome. Cooper et al. compared healthy people, and the subjects with low back pain were more likely to have weakened gluteus medius [27]. It was concluded from the study that people with gluteus medius weakness have lower back pain on the affected side, along with positive Trendelenburg signs [27]. An increase in the anterior pelvic tilt was seen due to tightness in hip flexors; there was a disturbance in the alignment of the lumbar spine, leading to postural changes; and weak lower abdominal muscles could lead to other complications if left untreated. Lower cross syndrome can disturb the kinetic chain of the lower limb, particularly the hip, knee, and trunk.

In a study by Helewa et al., the effects of back education and abdominal muscle strengthening exercises were compared in 402 asymptomatic patients in a randomized controlled trial [28]. The findings showed that there were no group differences in low back pain episodes, which may have been caused by noncompliance with the exercise regimen [28]. The largest prevalence of low back pain was seen in women and those between the ages of 40 and 80 years, according to a 2012 comprehensive analysis by Hoy et al. [29]. The estimated mean ± SEM point prevalence, after accounting for methodologic variation, was 11.9 ± 2.0% [29]. According to a a cross-sectional study by Mondal et al. on the prevalence of tightness in piriformis in healthy sedentary individuals, adult populations are more likely to experience piriformis muscle tightness, which can subsequently result in the development of piriformis syndrome and low back pain [30]. When Park et al. compared different postures with different amounts of time spent sitting with their legs crossed, they discovered that sitting with crossed legs for more than three hours a day can lead to various postural changes [31].

The above study was conducted on housemaids with low back pain, which gives us an idea of tightness and weakness leading to disturbance in the kinematic chain of the lower extremity. Ignorance of symptoms could further lead to chronic musculoskeletal problems, ultimately affecting their ADLs and productivity at work. The limitations of the above study are that the sample size was less, and objective measures which include diagnostic imaging were not used to confirm the presence of lower cross syndrome in the study. Specific ergonomic factors like household chores and duration of work shifts were not specified, and housemaids who complains of severe musculoskeletal issues may have been more likely to participate, leading to overestimation of the prevalence of lower cross syndrome in this population. More studies should be done on managing the symptoms of pain and ergonomic advice to improve productivity at work and overall quality of life.

## Conclusions

The conclusion of the above study, which aims to find the prevalence of lower cross syndrome in housemaids, showed positive results, including tightness and weakness of the segments of the muscles leading to an imbalance. It was concluded that the chances of the occurrence of the lower cross syndrome are common in females between the ages of 35 and 50 years. Tightness of the hip flexors and lumbar extensors and weak abdominal muscles were found in housemaids. This research suggests that housemaids working continuously for more hours without maintaining good ergonomic management are prone to lower cross syndrome. This indicates a strong need for further research into such a population (housemaids) to prevent chronic musculoskeletal illness. As a result, the study should be done on a large scale to find better outcomes in a population that always ignores their health due to their workload.
